# Single dose of BNT162b2 mRNA vaccine against severe acute respiratory syndrome coronavirus‐2 (SARS‐CoV‐2) induces neutralising antibody and polyfunctional T‐cell responses in patients with chronic myeloid leukaemia

**DOI:** 10.1111/bjh.17568

**Published:** 2021-06-03

**Authors:** Patrick Harrington, Katie J. Doores, Deepti Radia, Amy O’Reilly, Ho Pui Jeff Lam, Jeffrey Seow, Carl Graham, Thomas Lechmere, Donal McLornan, Richard Dillon, Yogita Shanmugharaj, Andreas Espehana, Claire Woodley, Jamie Saunders, Natalia Curto-Garcia, Jennifer O'Sullivan, Kavita Raj, Shahram Kordasti, Michael H. Malim, Claire Harrison, Hugues de Lavallade

**Affiliations:** ^1^ Department of Clinical Haematology Guy’s and St Thomas’ NHS Foundation Trust London UK; ^2^ School of Cancer and Pharmaceutical Science King’s College London London UK; ^3^ Department of Infectious Diseases School of Immunology and Microbial Sciences King’s College London London UK; ^4^ Department of Medicine and Molecular Genetics King’s College London London UK; ^5^ Department of Haematological Medicine King’s College London School of Medicine London UK

**Keywords:** SARS‐CoV2 vaccine, BNT162b2 vaccine, chronic myeloid leukaemia, tyrosine kinase inhibitor

## Abstract

Patients receiving targeted cancer treatments such as tyrosine kinase inhibitors (TKIs) have been classified in the clinically extremely vulnerable group to develop severe acute respiratory syndrome coronavirus‐2 (SARS‐CoV‐2), including patients with chronic myeloid leukaemia (CML) taking TKIs. In addition, concerns that immunocompromised individuals with solid and haematological malignancies may not mount an adequate immune response to a single dose of SARS‐CoV‐2 BNT162b2 (Pfizer‐BioNTech) vaccine have been raised. In the present study, we evaluated humoral and cellular immune responses after a first injection of BNT162b2 vaccine in 16 patients with CML. Seroconversion and cellular immune response before and after vaccination were assessed. By day 21 after vaccination, anti‐Spike immunoglobulin G was detected in 14/16 (87·5%) of the patients with CML and all developed a neutralising antibody response [serum dilution that inhibits 50% infection (ID_50_) >50], including medium (ID_50_ of 200–500) or high (ID_50_ of 501–2000) neutralising antibodies titres in nine of the 16 (56·25%) patients. T‐cell response was seen in 14/15 (93·3%) evaluable patients, with polyfunctional responses seen in 12/15 (80%) patients (polyfunctional CD4^+^ response nine of 15, polyfunctional CD8^+^ T‐cell response nine of 15). These data demonstrate the immunogenicity of a single dose of SARS‐CoV‐2 BNT162b2 vaccine in most patients with CML, with both neutralising antibodies and polyfunctional T‐cell responses seen in contrast to patients with solid tumour or lymphoid haematological malignancies.

## Introduction

Severe acute respiratory syndrome coronavirus‐2 (SARS‐CoV‐2), a novel beta coronavirus, has led to unprecedented healthcare challenges on a global scale. Development of anti‐viral immunity is key to reducing spread of infection and gaining pandemic control. Impressive collaborative efforts have led to the rapid development of multiple efficacious vaccines against SARS‐CoV‐2. BNT162b2 is a nucleoside‐modified mRNA that encodes a full‐length Spike that is stabilised in the pre‐fusion conformation of the SARS‐CoV‐2 Spike (S) protein, a key target of neutralising antibodies. However, concerns that immunocompromised individuals may not mount an adequate immune response after a single dose of vaccination have been raised, with early reports describing a reduced response in a heterogeneous group of patients with solid and haematological malignancies.^1^ Patients with chronic myeloid leukaemia (CML) have been shown to have impaired innate and adaptive immunity, although most pronounced at diagnosis, leading to the decision of the Department of Health and Social Care (DHSC) to classify in the clinically extremely vulnerable groups ‘people having other targeted cancer treatments that can affect the immune system, such as protein kinase inhibitors’. Immune response to vaccination may indeed be attenuated by tyrosine kinase inhibitors (TKIs), particularly those with greater ‘off target’ kinase inhibition, which we have previously shown to inhibit B‐cell function and antibody response *in vivo*.^2^ In the present study, we report an analysis of the T‐cell and antibody responses following a single dose of BNT162b2 in patients with CML on TKI therapy.

## Methods

### Study design and vaccine

From 17 December 2020 until 17 February 2021, patients with a known diagnosis of myeloid haematological malignancies presenting at Guy’s and St Thomas’ NHS Foundation Trust and eligible to receive a 30 μg injection of SARS‐CoV‐2 mRNA BNT162b2 vaccine were approached for informed consent. Over that period, 16 adult patients with a diagnosis of chronic phase CML were vaccinated in compliance with UK DHSC guidelines and recruited. All patients gave informed consent and the study protocol was approved by the Regional Research and Ethics Review Board [Integrated Research Application System (IRAS) Identification (ID): 285396; Research Ethics Committee (REC) ID: 20/WM/0187]. Peripheral blood mononuclear cells (PBMCs) and plasma were isolated before vaccination and at a median [interquartile range (IQR)] of 21 [21–27·25] days after their first injection of 30 μg BNT162b2 in the 16 patients.

### Safety assessments

We solicited reports of local (pain, tenderness, redness, induration and ecchymosis) and systemic (fever, headache, malaise, myalgia, chills and nausea) adverse events by 2‐weekly telephone calls performed by trained medical students and physician assistants. All local and systemic adverse events reported in response to solicitation within 7 days after administration of the vaccine were considered to be related to the vaccine.

### Intracellular cytokine flow cytometry assay

T‐cell functionality was assessed using intracellular cytokine staining after incubation with SARS‐CoV‐2 specific peptides covering the immunogenic domains of the S protein (Miltenyi Biotech, Bergisch Gladbach, Germany). Briefly, cells were thawed and then rested for 18 h at 37°C, 5% CO_2_. Specific peptides (0·25 µg/ml) and anti‐cluster of differentiation (CD)28 (BD Biosciences, San Jose, CA, USA) were added for 3 h, followed by Brefeldin‐A (BFA) for an additional 3 h. Unstimulated cells were utilised as negative controls and phorbol myristate acetate (PMA) and Ionomycin (Miltenyi Biotech) was added separately as a positive control. Cells were stained with a viability dye, stained with antibodies directed against surface markers, and fixed and permeabilised with CytoFix/Cytoperm (BD Biosciences), before staining with antibodies directed against intracellular cytokines. Directly conjugated monoclonal antibodies with the following specificities were used; CD3 BUV395 (clone SK37), CD4 phycoerythrin (PE; clone M‐T477), CD45RO BV711, tumour necrosis factor alpha (TNF‐α; clone MAB11), interferon gamma (IFN‐γ) allophycocyanin (APC; clone B27), interleukin 2 (IL‐2) PE (clone MQ1‐17H12). Live/dead staining was performed using Zombie NIR™ amine reactive fluorescent dye (BioLegend, San Diego, CA, USA). Gating on the lymphocyte population, single cells, live cells, CD3^+^ cells, CD4^+^ cells and CD4^−^ (CD8^+^) cells was performed. T‐cell analysis was performed on a BD Fortessa cytometer and results processed using FlowJo, version 10·5 (FlowJo LLC, Ashland, OR, USA). Statistical analysis was performed using GraphPad Prism, version 8 (GraphPad Software Inc., San Diego, CA, USA).

### Enzyme‐linked immunosorbent assay (ELISA) protocol

The ELISAs were conducted as previously described.^3^ All plasma samples were heat‐inactivated at 56°C for 30 min before use. High‐binding ELISA plates (Corning, 3690; Corning, NY, USA) were coated with antigen Nuclear (N) protein or the S glycoprotein at 3 µg/ml (25 µl/well) in phosphate‐buffered saline (PBS), either overnight at 4°C or for 2 h at 37°C. Wells were washed with PBS‐T (PBS with 0·05% Tween‐20) and then blocked with 100 µl of 5% milk in PBS‐T for 1 h at room temperature. The wells were emptied and serial dilutions of plasma (starting at 1:25, sixfold dilution) were added and incubated for 2 h at room temperature. Control reagents included CR3009 (2 µg/ml; N‐specific monoclonal antibody), CR3022 (0·2 µg/ml; S‐specific monoclonal antibody), negative control plasma (1:25 dilution), positive control plasma (1:50) and blank wells. Wells were washed with PBS‐T. Secondary antibody was added and incubated for 1 h at room temperature. Immunoglobulin G (IgG) was detected using goat anti‐human‐Fc‐alkaline phosphatase (AP; 1:1000; Jackson ImmunoResearch Europe Ltd, Ely, Cambridgeshire, UK; catalogue no. 109‐055‐098) and wells were washed with PBS‐T and AP substrate (Sigma‐Aldrich, St. Louis, MO, USA) was added and plates read at 405 nm. The half maximal effective concentration (EC_50_) values were calculated in GraphPad Prism. Where an EC_50_ was not reached at 1:25, plasma was considered seropositive if the optical density (OD) at 405 nm was fourfold above background and a value of 25 was assigned.

### Neutralisation assay with SARS‐CoV‐2

Human immunodeficiency virus type‐1 (HIV‐1)‐based virus particles, pseudotyped with SARS‐CoV‐2 Wuhan Spike were prepared in HEK‐293T/17 cells and neutralisation assays were conducted as previously described.^3^ Serial dilutions of plasma samples (heat inactivated at 56°C for 30 min) were prepared in Dulbecco’s modified Eagle’s medium (DMEM) complete media [10% fetal bovine serum, 1% penicillin/streptomycin (100 iu/ml penicillin and 100 mg/ml streptomycin)] and incubated with pseudotyped virus for 1 h at 37°C in 96‐well plates. Next, HeLa cells stably expressing the angiotensin‐converting enzyme 2 (ACE2) receptor (provided by Dr James Voss, Scripps Research, La Jolla, CA, USA) were added (12 500 cells/50 µl per well) and the plates were left for 72 h. Infection level was assessed in lysed cells with the Bright‐Glo luciferase kit (Promega Corp., Madison, WI, USA), using a Victor™ X3 multi‐label reader (PerkinElmer, Inc., Waltham, MA, USA). Measurements were performed in duplicate and the duplicates used to calculate the serum dilution that inhibits 50% infection (ID_50_) using GraphPad Prism.

## Results

### Patients’ characteristics and toxicity profile of SARS‐CoV‐2 BNT162b2

Analysis was performed in the 16 patients with chronic phase CML, with clinical characteristics summarised in Table I. The vaccine was safe and tolerable in this patient group with localised inflammation reported in 56·3% (nine) and a transient flu‐like illness reported in 23·5% (four) of patients (Table II). None of the 16 patients reported SARS‐CoV‐2 infection before enrolment or after inclusion in the study.

**Table I:** Patient’s baseline characteristics and response to first dose of vaccination.

Pt.	Age, years	Sex	Ethnicity	TKI	Dose, mg	Response	Pre CD4	Pre CD8	Post CD4	Post CD8	Pre anti‐N IgG OD	Pre anti‐S IgG EC_50_	Post anti‐S IgG EC_50_	Post neutralising Ab ID_50_
01	37	F	Caucasian	Ponatinib	15	MR4·5	NA	NA	NA	NA	0·175	<25	224	714
02	62	F	Caucasian	Imatinib	400	MR4·5	−	−	+	−	0·272	<25	25	190
03	50	M	Caucasian	Nilotinib	800	MMR	−	−	−	+	0·228	<25	32	804
04	23	M	Caucasian	Nilotinib	600	<CCyR	−	−	+	+	0·181	<25	158	467
05	74	M	Caucasian	Ponatinib	15·alt.	<CCyR	−	−	−	+	0·221	<25	25	116
06	36	M	Black‐African	Imatinib	400	MR4·5	NA	NA	+	−	0·366	25	1421	1·122
07	43	M	Black‐African	Imatinib	400	MMR	NA	NA	+	+	0·203	<25	172	511
08	48	M	Caucasian	Bosutinib	300	MMR	+	−	+	+	0·176	<25	759	1·489
09	26	F	Caucasian	Dasatinib	100	CCyR	−	−	+	+	0·195	<25	722	586
10	46	M	Caucasian	Ponatinib	15	CCyR	NA	NA	+	−	0·193	<25	<25	50
11	74	M	Caucasian	Bosutinib	100	MMR	−	−	+	−	0·165	<25	<25	65
12	55	F	Black‐British	Imatinib	400	MR4·5	−	−	+	+	0·18	<25	331	424
13	47	M	Caucasian	Dasatinib	100	MMR	NA	NA	+	−	0·18	<25	25	165
14	36	M	Caucasian	Nilotinib	600	MR4·5	NA	NA	+	+	0·201	<25	43	140
15	37	M	Caucasian	Nilotinib	300	MR4·5	NA	NA	+	+	NA	NA	434	479
16	36	M	Caucasian	Ponatinib + HSCT	30	MR4·5	NA	NA	−	−	NA	NA	25	89

Ab, antibody; CCyR, complete cytogenetic response; CD, cluster of differentiation; EC_50_, 50% effective concentration; F, female; ID_50_, 50% inhibitory dilution; IgG, immunoglobulin G; HSCT, haematopoietic stem cell transplant; M, male; MMR, major molecular response; MR4·5, 4·5 log molecular response; NA, not applicable; OD, optical density; Pt., patient number; TKI, tyrosine kinase inhibitor.

**Table II:** Injection‐site and systemic adverse effects after receiving the vaccine among patients.

Patient number	Age, years	Adverse event	
		Local	Systemic
01	37	None	None
02	62	None	Flu‐like illness
03	50	Localised inflammation	None
04	23	Localised inflammation	Fatigue
05	74	Localised inflammation	None
06	36	Localised inflammation	None
07	43	None	Flu‐like illness Headache
08	48	Localised inflammation	Flu‐like illness
09	26	None	Fatigue
10	46	None	Flu‐like illness
11	74	Localised inflammation	None
12	55	None	None
13	47	None	None
14	36	Localised inflammation	None
15	37	None	Flu‐like illness
16	36	Localised inflammation	Headache

### Seroconversion rates to BNT162b2 and neutralising antibody titres after a first injection

**Fig 1:**
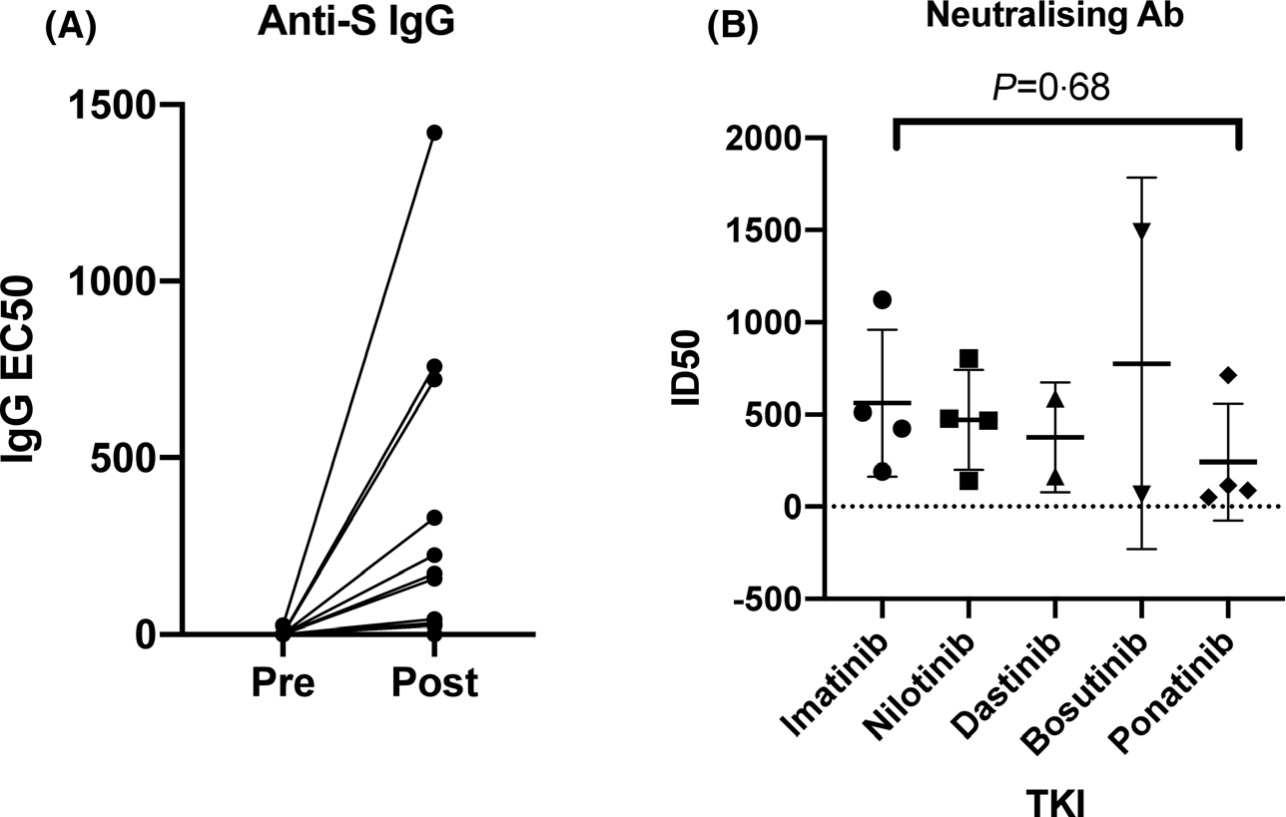
(A) Anti‐S IgG EC50 pre and post vaccination. (B) Neutralising antibody ID50 within different TKI groups. EC50, 50% effective concentration; ID50, 50% inhibitory dilution; TKI, tyrosine kinase inhibitor.

Antibody testing was performed in all 16 patients with CML. The presence of IgG to Spike and plasma neutralising activity was measured 3 weeks after the first vaccine injection [median (IQR) 21 (21–27·25) days]. A positive anti‐S IgG ELISA response was seen in 87·5% (14/16) of patients analysed after a first injection, with only two patients showing undetectable IgG to Spike at 1:25 dilution (Table I and Fig 1A). Of the two patients with a negative serological response, one had previously been investigated for potential immune suppression and recurrent infections, whilst the other was on second‐line treatment with ponatinib, with suboptimal response.

The median (IQR) EC_50_ value for anti‐S IgG in all patients from ELISA testing was 100 5 (25–408·3). Amongst different TKIs the median EC_50_ values were 251 5 for imatinib, 100·5 for nilotinib, 373·5 for dasatinib, 379·5 for bosutinib and 25 for ponatinib. No patients had evidence of confirmed previous infection as determined by a >fourfold increase in OD of IgG against the Nucleocapsid (N) and S protein in the pre‐vaccine sample.^4^ However, a single patient did have a weakly positive anti‐S IgG EC_50_, as well as a mildly raised anti‐N OD on a pre‐vaccine sample, suggestive of possible previous infection.

Neutralising antibody analysis was performed in all post‐vaccine samples using an HIV‐1‐based virus particles, pseudotyped with SARS‐CoV‐2 Wuhan Spike, with positive responses seen in all patients and a median (IQR) ID_50_ of 445 5 (122–682). Seven of the 16 (43·8%) patients showed low (50–200), three (18·8%) medium (201–500) and six (37·5%) high (501–2 000) neutralising titres.^3^Amongst different TKIs the median ID_50_ was 467 5 for imatinib, 473 for nilotinib, 375·5 for dasatinib, 777 for bosutinib and 102·5 for ponatinib, with no statistical difference seen between TKIs (Fig 1B, *P* = 0·68). Moreover, analysis was performed after both first and second doses in one patient (Patient 01), with an increase in IgG EC_50_ from 224 to 4810 and neutralising antibody ID_50_ increase from 714 to 2037, between the first and second doses.

### T‐cell response to first injection of BNT162b2

The induction of virus‐specific T‐cell responses by BNT162b2 vaccination was assessed directly *ex vivo* by flow cytometric enumeration of antigen‐specific CD8^+^ and CD4^+^ T lymphocytes using an intracellular cytokine assay for IFN‐γ, TNF‐α and IL‐2. Before vaccination, T‐cell analysis was performed in eight patients, with only one patient showing evidence of a monofunctional CD4^+^ T‐cell response to the S protein with expression of IFN‐γ (Table I).

**Fig 2:**
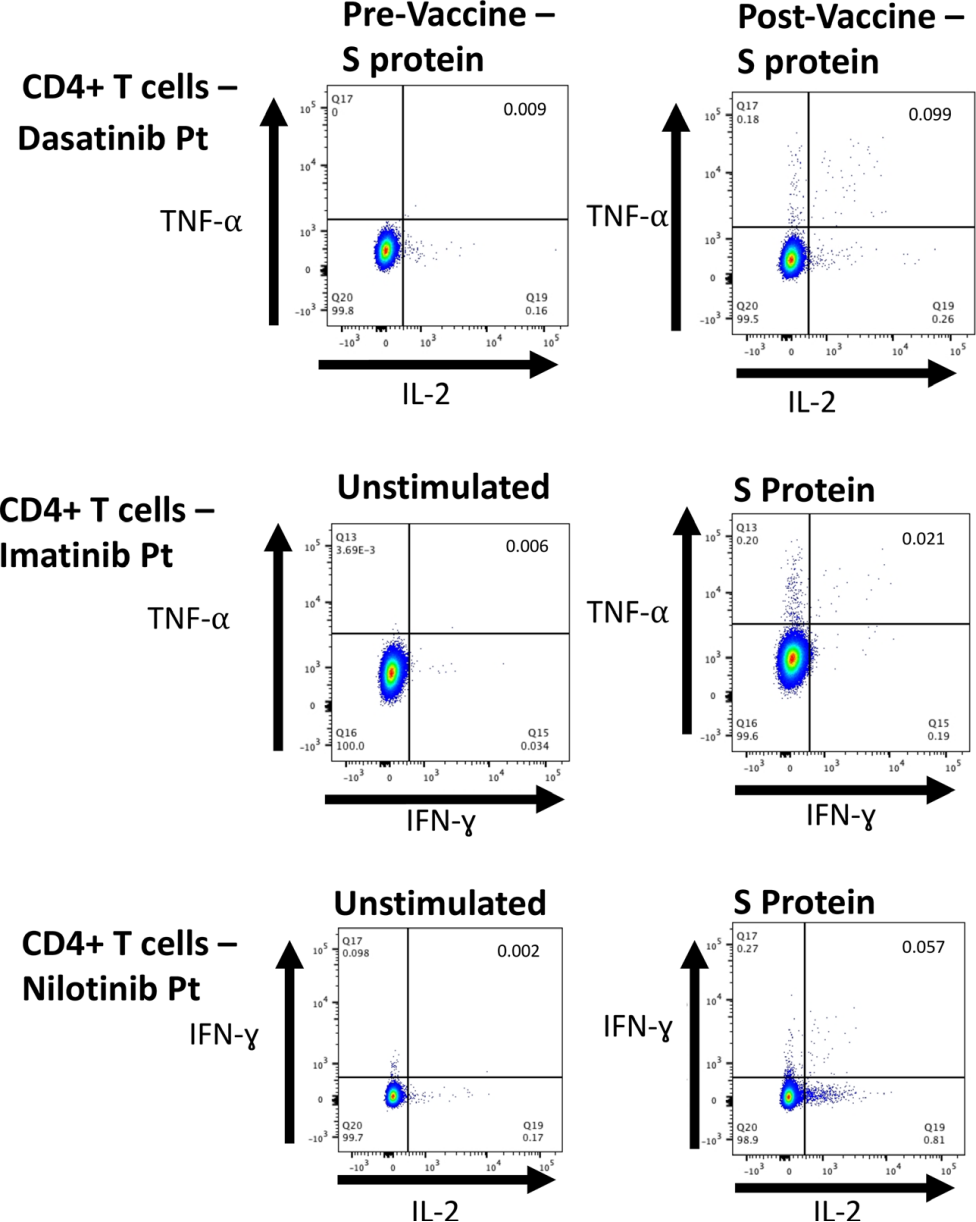
Top: dual expression of TNF alpha (TNF‐α) and interleukin 2 (IL‐2) in CD4+ T cells in dasatinib treated patient. Left, pre‐vaccine; and right, post vaccine. Middle: dual expression of TNF‐α and interferon gamma (IFN‐γ) in CD4+ T cells in imatinib treated patient. Left, unstimulated cells; and right, cells exposed to S protein. Bottom: dual expression of IFN‐γ and IL‐2 in CD4+ T cells in nilotinib treated patient. Left, unstimulated cells; and right, cells exposed to S protein.

After a first injection of BNT162b2, T‐cell analysis was performed in 15 patients. A response was considered to be positive if there was a threefold increase in any pro‐inflammatory cytokine from baseline expression, and above a threshold of 0 01. A memory T‐cell response was seen in 14 out of 15 evaluable patients (93·3%), with the only patient not showing a T‐cell response being post allogeneic haematopoietic stem cell transplantation (HSCT) and taking ponatinib (Table I and Fig 2). A SARS‐CoV‐2 specific CD4^+^ T‐cell response was seen in 80% (12/15) and a SARS‐CoV‐2 specific CD8^+^ T‐cell response was seen in 60% (nine of 15, Table I and Fig 2). A polyfunctional cytokine response in either CD4 or CD8^+^ T cells was seen in 80% (12/15) of patients, with a polyfunctional CD4^+^ response in 60% (nine of 15) and a polyfunctional CD8^+^ T‐cell response in 40% (six of 15) (Fig 2).

**Fig 3:**
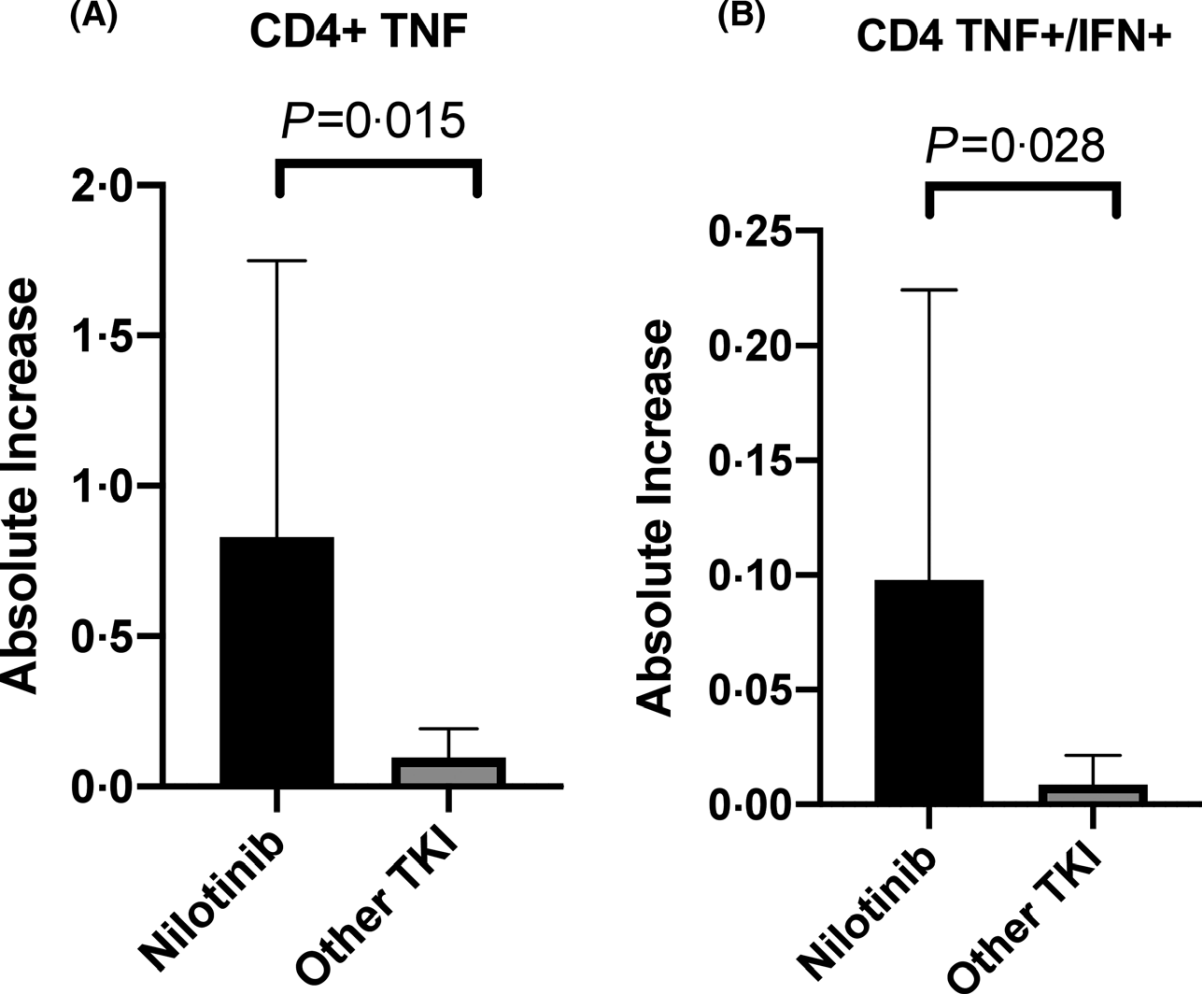
(A) Mean increase in CD4+ T cell TNF alpha (TNF‐α) expression in nilotinib‐treated patients compared with other tyrosine kinase inhibitors (TKIs). (B) Mean increase in CD4+ T cell dual TNF‐α and interferon gamma (IFN‐γ) expression in nilotinib‐treated patients compared with other TKIs.

The median (IQR) increase in expression of TNF‐α in CD4^+^ cells compared with the baseline unstimulated control was 0 071 (0·039–0 25) and in CD8^+^ cells 0 032 (0·004–0·6). The median (IQR) expression of IFN‐γ was 0·027 (0–0·11) in CD4^+^ and 0·091 (0·007–1·18) in CD8^+^ cells, whilst the median (IQR) IL‐2 expression was 0·05 (0·05–0·11) in CD4^+^ cells and 0·01 (0·056–0·09) in CD8^+^ cells. With regards to polyfunctional responses, the median (IQR) increase in TNF‐α^+^/IFN‐γ^+^ cells was 0·004 (0·002–0·014) in CD4^+^ cells and 0·007 (0–0·039) in CD8^+^ cells, with a median (IQR) increase in expression of TNF‐α^+^/IL‐2^+^ of 0·05 (0·05–0·11) in CD4^+^ cells and 0·002 (0–0·004) in CD8^+^ cells. More than 90% of reactive cells expressing IFN‐γ or TNF‐α co‐expressed CD45RO, consistent with a memory T cell phenotype. Interestingly patients taking nilotinib had a significantly higher mean increase in TNF‐α expression in CD4^+^ cells, when compared with patients taking other TKIs (0·83 vs. 0·096, *P* = 0·015; Fig 3A), and also of dual TNF‐α^+^/IFN‐γ^+^ (0·098 vs. 0·009, *P* = 0·028; Fig 3B). No other significant differences between TKIs were identified.

## Discussion

Large international randomised placebo‐controlled studies have shown the efficacy of the BNT162b2 vaccine, with a two‐dose regime resulting in a 95% reduction in cases in the general population.^5^ Evidence of a protective immune response to a single dose is shown by a reduction in cases of 52% reported in the interim between first and second doses in those receiving BNT162b2.^5^ Sahin *et al*.^6^ reported a detailed analysis of the immune response to BNT162b2 in healthy controls, with a Th1 skewed CD4^+^ T‐cell response in 95·2% and a CD8^+^ T‐cell response in 76·2%, as determined by Elispot testing. They also described antibody responses to vaccination that were significantly greater than those observed in a group of patients who had recovered from SARS‐CoV‐2 infection.^6^

T‐cell immunity may prove to be particularly important for protection from SARS‐CoV‐2, with the evidence from the SARS‐CoV‐1 epidemic showing that antibody responses were relatively short‐lived following infection, whilst memory T‐cell responses were significantly more durable.^7^ Our group has recently reported a significant decline in neutralising antibody levels in the 3 months after infection with SARS‐CoV‐2.^3^ We have also reported that robust T‐cell responses can be observed as long as 6 months after infection with SARS‐CoV‐2 in a small cohort of patients with chronic myeloproliferative neoplasms including CML.^8^ Furthermore, a number of additional studies have elegantly demonstrated the T‐cell response to SARS‐CoV‐2 in the general population.^9^, ^10^, ^11^, ^12^, ^13^

However, a recently submitted report describes a significantly reduced immune response in a heterogeneous group of patients with solid and haematological malignancies,^1^ bringing concerns over the ability of clinically vulnerable patients with haematological malignancies to mount a protective response following a single dose of SARS‐CoV‐2 BNT162b2 vaccination.

Among patients with chronic myeloid malignancies, TKI therapy is recognised to cause variable immune deficiency, with TKIs that also inhibit Src family kinases causing significant T‐cell dysfunction due to the pivotal role that these kinases play in signalling downstream from the T‐cell receptor.^14^ We have previously shown that B‐cell function is also impaired by TKI therapy through off target inhibition of Btk, a kinase that is important for normal B‐cell signalling, leading to reduced efficacy of the seasonal influenza vaccination.^2^ However, during the influenza A virus subtype H1N1 (H1N1) vaccination campaign, we demonstrated that the seroprotection rate to a first injection of 2009 H1N1 vaccine [Pandemrix; GlaxoSmithKline (GSK) plc, Brentford, Middlesex, UK] was not significantly different between patients with chronic phase CML and healthy controls, unlike other haematological conditions such as B‐cell malignancies or recipients of HSCT.^15^

Consistent with our previous experience in H1N1 and seasonal influenza vaccination, our data show encouraging results, with 87·5% seroconversion to a first SARS‐CoV‐2 BNT162b2 injection in patients with CML, contrasting with what has been described in the first report of vaccination in patients with cancer.^1^ In addition, nearly all the patients with CML in our present analysis demonstrated both mono‐ and polyfunctional T‐cell cytokine responses to SARS‐CoV‐2, a response that is similar to healthy controls tested in previous trials of BNT162b2 vaccination,^6^ although it should be noted that methodology differs between studies. Overall, both seroconversion and T‐cell response to a single injection demonstrate the immunogenicity of SARS‐CoV‐2 BNT162b2 in patients with CML, with neutralising antibodies responses seen in all patients. To our knowledge the present report is the first of humoral and T‐cell response to vaccination against SARS‐CoV‐2 in patients with CML taking TKIs. These data are particularly important in the context of the decision from the DHSC to increase the interval between a first and a second vaccine injection, and demonstrate that patients with CML taking TKIs receive significant protection from a first injection of SARS‐CoV‐2 BNT162b2.

Finally, we found that patients taking nilotinib had a significantly greater increase in TNF‐α and dual TNF‐α/IFN‐γ expression in SARS‐CoV‐2 specific CD4^+^ cells, when compared with patients taking other TKIs. This suggests that the highly selective ABL1 TKI nilotinib, which does not inhibit Src kinases or other kinases important to the immune response, may have less effect on the patients’ immune system. However, it is not possible to draw definitive conclusions on the effect of different TKIs from this relatively small cohort.

Further prospective data are required to confirm that the immune response to vaccination we have described in the present study translates into a reduction in SARS‐CoV‐2 infections in this patient group. In addition, longitudinal studies are required to characterise the response to the two‐dose vaccination regimen, as a second injection may provide a stronger and longer lasting protection. Additional studies will need to determine if the immune response will be sufficient to protect against emerging variants, as well as analyse the effect of other currently available and emerging vaccines against SARS‐CoV‐2.

## Funding

King’s Together Rapid COVID‐19 Call awards to Michael H. Malim, Katie J. Doores; A Huo Family Foundation Award to Michael H. Malim, Katie J. Doores; Chronic Disease Research Foundation award CDRF‐22/2020 to Katie J. Doores, Michael H. Malim; Wellcome Trust Investigator Award 106223/Z/14/Z to Michael H. Malim; Carl Graham was supported by the MRC‐KCL Doctoral Training Partnership in Biomedical Sciences (MR/N013700/1); Fondation Dormeur, Vaduz for funding equipment to Katie J. Doores. Blood Cancer UK (funding for Hugues de Lavallade); LifeArc (funding for Shahram Kordasti); Cancer Research UK (CRUK), King's Health Partners Centre (funding for Shahram Kordasti).
